# FOXA1, GATA3 and PPARɣ Cooperate to Drive Luminal Subtype in Bladder Cancer: A Molecular Analysis of Established Human Cell Lines

**DOI:** 10.1038/srep38531

**Published:** 2016-12-07

**Authors:** Joshua I. Warrick, Vonn Walter, Hironobu Yamashita, Eunah Chung, Lauren Shuman, Vasty Osei Amponsa, Zongyu Zheng, Wilson Chan, Tiffany L. Whitcomb, Feng Yue, Tejaswi Iyyanki, Yuka I. Kawasawa, Matthew Kaag, Wansong Guo, Jay D. Raman, Joo-Seop Park, David J. DeGraff

**Affiliations:** 1 Department of Pathology, Pennsylvania State University Milton S. Hershey Medical Center, Hershey, PA, USA; 2 Department of Surgery, Division of Urology, Pennsylvania State University College of Medicine, PA, USA; 3 Department of Biochemistry and Molecular Biology, Pennsylvania State University College of Medicine, PA, USA; 4 Department of Public Health Sciences, Pennsylvania State University College of Medicine, PA, USA; 5 Division of Pediatric Urology and Developmental Biology, Cincinnati Children’s Hospital Medical Center, OH, USA; 6 Institute for Personalized Medicine, Pennsylvania State University College of Medicine, PA, USA; 7 Department of Surgery, Division of Urology, Changchun Central Hospital, Changchun, China; 8 Department of Comparative Medicine, Pennsylvania State University College of Medicine, PA, USA.

## Abstract

Discrete bladder cancer molecular subtypes exhibit differential clinical aggressiveness and therapeutic response, which may have significant implications for identifying novel treatments for this common malignancy. However, research is hindered by the lack of suitable models to study each subtype. To address this limitation, we classified bladder cancer cell lines into molecular subtypes using publically available data in the Cancer Cell Line Encyclopedia (CCLE), guided by genomic characterization of bladder cancer by The Cancer Genome Atlas (TCGA). This identified a panel of bladder cancer cell lines which exhibit genetic alterations and gene expression patterns consistent with luminal and basal molecular subtypes of human disease. A subset of bladder cancer cell lines exhibit *in vivo* histomorphologic patterns consistent with luminal and basal subtypes, including papillary architecture and squamous differentiation. Using the molecular subtype assignments, and our own RNA-seq analysis, we found overexpression of GATA3 and FOXA1 cooperate with PPARɣ activation to drive transdifferentiation of a basal bladder cancer cells to a luminial phenotype. In summary, our analysis identified a set of human cell lines suitable for the study of molecular subtypes in bladder cancer, and furthermore indicates a cooperative regulatory network consisting of GATA3, FOXA1, and PPARɣ drive luminal cell fate.

Urothelial carcinoma (UC) of the bladder is the second most common urologic malignancy, accounting for over 70,000 new cancer diagnoses in the United States each year^1^. Through the discovery of intrinsic molecular subtypes of bladder UC with particular phenotypic characteristics, several research groups have revolutionized our understanding of this common malignancy^2^,^3^,^4^,^5^,^6^,^7^. For example, the “luminal” subtype comprises the majority of early stage (non-invasive) bladder cancers, as well as a significant fraction of muscle invasive disease. This subtype is enriched for papillary histomorphology^4^. The “basal” subtype often exhibits squamous differentiation, is biologically aggressive, and is found almost exclusively in invasive disease^4^,^8^. Evidence suggests that putative markers and transcriptional regulators of urothelial differentiation are upregulated in the luminal molecular subtype and downregulated in the basal subtype of bladder cancer^2^,^3^,^4^,^5^,^9^,^10^,^11^,^12^,^13^,^14^,^15^,^16^,^17^,^18^,^19^,^20^. However, there is limited experimental data (with some notable exceptions^2^,^21^) identifying a direct contribution of these transcription factors to molecular subtype. Indeed, an incomplete understanding of the suitability of available models to test the direct contribution of specific factors to the establishment of a given molecular subtype in UC is a significant hurdle to experimental design. Moreover, the optimal approach for analysis of gene data derived from genetic analysis is not always clear. Therefore, our research group undertook a detailed analysis of publically available gene expression, copy number alteration, and mutational data for 27 bladder UC cell lines available through the Cancer Cell Line Encyclopedia (CCLE^22^), and applied analyses similar to those performed in The Cancer Genome Atlas (TCGA^4^) study. This approach identified a subset of cell lines as suitable models for the study of molecular subtypes of bladder cancer. In conjunction with other published studies^23^, this will provide an important resource for the bladder cancer research community. As previous research indicates transcriptional regulation is associated with molecular subtype in bladder cancer^12^, we additionally used our approach to directly test the ability of the transcription factors GATA3 and FOXA1, which are consistently associated with the luminal molecular subtype of UC, to cooperate with activation of PPARγ to drive the luminal molecular subtype.

## Materials and Methods

### Urothelial Cancer Cell Line Classification

Bladder UC cell lines (n = 27) were classified into molecular subtypes using publically available CCLE data, in a manner similar to TCGA bladder cancer analysis^4^. Cell line expression subtypes were identified and defined using the TCGA expression subtype gene list and agglomerative methods (see supplemental material for complete protocol). Selected alterations in genes identified as significantly mutated or with significant copy number alterations (CNAs) in the TCGA study were assessed in CCLE UC cell lines (see supplemental protocol), and compared to assigned expression subtypes.

### Tissue Recombination Xenografting

All animal experiments were performed in accordance with approved guidelines of the Institutional Animal Care and Use Committee (IACUC) of the Pennsylvania State University College of Medicine. Additionally, all experimental protocols were approved by the Pennsylvania State University College of Medicine IACUC. Isolation of embryonic bladder mesenchyme (eBLM), preparation of tissue recombinants, and kidney capsule surgeries were performed as described previously^10^,^18^. Pregnant rats (Harlan Laboratories, Tampa FL) were sacrificed at embryonic day 16 (E16) (plug day = 0). Embryos were isolated as previously described^24^, and bladders were microdissected under dissecting microscope (Olympus SZX7, Waltham MA) from isolated embryos, and embryonic bladders were separated from the urogenital sinus at the bladder neck and the attached ureters carefully dissected using Vanna spring scissors (Fine Science Tools, Foster City CA). Whole bladders were then placed into calcium and magnesium-free Hanks’ saline (Gibco) containing 25 mM EDTA (Sigma, St. Louis MO) for 90 min at room temperature to release the bladder urothelium. The mesenchyme and urothelium were separated manually under microscopic examination using two 25 gauge needles connected to a 1cc syringe, leaving the mesenchyme behind as a bladder shell. RT4 (50,000), UMUC1 (1 × 10^5^), and SCaBER (1 × 10^5^) cells were re-suspended in 50 microliters of a 3∶1 ratio of rat tail collagen and setting solution, and were plated in 10 cm dishes. Following the insertion of 1 eBLM per aliquot, tissue recombinants were placed at 37 °C to promote solidification. McCoy’s modified medium (ThermoFisher Scientific, Grand Island NY) containing 10% FBS was then applied to solidified grafts and incubated overnight. The following day, tissue recombinants were placed under the kidney capsule of the left kidney of 5 SCID mice, resulting in a total of 10 grafts for each cell line. Three weeks following implantation, mice bearing RT4 cells were sacrificed, whereas mice bearing UMUC1 and SCaBER cells were sacrificed 1 and 2 months after surgery, respectively. Dissected kidneys containing tissue recombinants were fixed in formalin and subjected to standard processing in preparation for immunohistochemistry.

### Immunohistochemistry

Immunohistochemistry (IHC) was performed as previously described^9^. Briefly, slides were deparaffinized and rehydrated through a series of graded alcohols and washed in deionized water for 5 minutes. Antigen retrieval was performed by placing slides in 1% antigen unmasking solution (Vector Labs, Burlingame, CA) and heating slides for 25 minutes on high power in a pressure cooker (Cuisinart CPC-600FR). Steam was released in short bursts to prevent boiling and preserve tissue integrity. Slides were cooled to room temperature and washed 3 times for 10 minutes in PBS (pH 7.4). All incubations were performed at room temperature unless otherwise noted. Endogenous peroxidases were blocked by incubation in 1% hydrogen peroxide in methanol for 20 minutes, and slides were again washed 3 times for 10 minutes in phosphate-buffered saline (PBS). Sections were incubated in PBS containing horse serum (Vector Labs) for 30 minutes to reduce nonspecific antibody binding and then incubated overnight with primary antibody at 4 °C in a humidified chamber. Primary antibodies used for IHC include goat anti FOXA1 (1:1000; Santa Cruz Biotechnology, Santa Cruz, CA), mouse anti GATA3 (1:200; Santa Cruz Biotechnology), pan UPK/AUM (1:20,000), rabbit anti UPK1A (1:2000), rabbit anti UPK1B (1:50), rabbit anti UPKII (1:1000). mouse anti UPKIIIA (1:300); (All a kind gift from Dr. Xue-Ru Wu at New York University), mouse anti cytokeratin 5/6 (KRT5/6; 1:200; Dako, Carpinteria, CA), mouse anti Cytokeratin 14 (KRT14; 1:200; Vector Laboratories, Burlingame CA), mouse anti Cytokeratin 20 (KRT20; 1:100; Abcam, Cambridge MA), and rabbit anti EGFR (1:200, Sigma). Following overnight incubation, slides were washed 3 times for 10 minutes in PBS and sections were incubated in biotinylated secondary antibody diluted in PBS containing horse serum (1:200; Vector Labs) for 1 hour. Specific antibody binding was visualized using Vectastain Elite ABC Peroxidase kit (Vector Labs) according to the manufacturer protocol with diaminobenzidine substrate buffer as the chromogen (Thermo Scientific).

### Cell Culture

All UC cell lines were purchased from the American Type Culture Collection (ATCC), except UMUC1 bladder cancer cells (Sigma Aldrich). The bladder cancer cell lines RT4^25^ and T24^26^ were cultured in McCoy’s Modified Medium with 10% FBS. UMUC1^27^ and UMUC3^28^ UC cells were cultured in Minimal Essential Medium supplemented with 10% FBS. SCaBER^29^, HT1197^30^, HT1376^30^ and TCCSUP^31^ UC cell lines were cultured in MEM following the addition of Non-Essential Amino Acids (NEAA) and 10% FBS. The UC cell lines 5637^32^ and SW780^33^ were cultured in RPMI 1640 following the addition of 10% FBS. All cell lines are routinely screened for mycoplasma infection (PromoKine, Heidelberg), and authenticated via STR analysis (Genetica, Burlington NC).

### Transient Transfection and PPARɣ Agonist Treatment

Transient transfection experiments were used to determine the impact of individual or combined FOXA1 and GATA3 expression on gene expression and molecular subtype assignment both in the presence and absence of the PPARɣ agonist rosiglitazone. The day before transfection, 2 × 10^5^ 5637 bladder cancer cells were plated in 6 well plates (Corning Inc, Corning, NY). The following day, attached cells were transfected with the following plasmid constructs: pCMV6-Entry (CMV empty vector; Origene, Rockville MD), pCMV6-FOXA1 (Origene; RC206045), pCMV6-GATA3 (Origene; RC211904), or a combination of pCMV6-FOXA1 and pCMV6-GATA3 using Lipofectamine 3000 (Life Technologies, Carlsbad CA), and incubated for 6 hours, after which medium containing DNA-lipid complex was removed and replaced with fresh Minimum Essential Medium (MEM)/Earle’s Balanced Salt Solution (EBSS) medium and incubated for an additional 48 hours to enable cell recovery. After 48 hours, culture medium was removed and washed once with serum-free MEM/EBSS medium, followed by the addition of serum-free MEM/EBSS medium to each well and incubated for 24 hours. After serum starvation, rosiglitazone (1 micromolar; TOCRIS, Bristol, UK) or Dimethyl sulfoxide (DMSO; Sigma; vehicle control) was added to transfected cells and cultured for an additional 48 hours. At the conclusion, RNA was extracted (RNeasy; Thermo Fisher Scientific) and cDNA was prepared by using the SuperScript VILO cDNA synthesis kit (Thermo Fisher Scientific) according to manufacturer’s protocol.

### Western Blotting

All cell lysates were prepared using RIPA lysis and extraction buffer (Thermo Fisher Scientific). Protein concentrations following cell lysis were measured by using the PierceBCA Protein Assay Kit (Thermo Fisher Scientific) as per manufacturer’s instructions. Following extraction, protein samples (40 μg of, 1x LDS sample buffer, 10% 2-mercaptoethanol) were electrophoresed on 4–12% Bis-Tris NuPAGE gels (Thermo Fisher Scientific), and proteins were subsequently transferred to nitrocellulose Blotting Membrane (GE Healthcare Life science, Fairfield CT) using a Pierce G2 Fast Blotter (Thermo Fisher Scientific) according to manufacturer’s protocol. Following transfer, membranes were incubated at room temperature in 5% non-fat milk (NFDM) dissolved in Tris buffered saline containing 0.1% Tween-20 (TBST) for 1 hour. Additionally, all primary antibodies used in this study were diluted in TBST with 5% NFDM. Dilutions of primary antibodies were as follows: anti-FOXA1 (1:500; ab23738, Abcam), anti-PPARɣ (2430 (D69), Cell Signaling technologies), anti-EGFR (D38B1; Cell Signaling Technologies; 1:1000), anti-KRT5/6 (Clone D5/16 B4; DAKO 1:200), and anti-GAPDH (14C10; Cell Signaling technologies). After incubation with primary antibodies overnight at 4 degrees Celsius, all membranes were washed 5 times for 5 minutes with TBST. Secondary antibody (ECL anti-rabbit or mouse IgG, HRP-linked whole antibody; 1:2000; GE healthcare Life science) was diluted in TBST containing 5% NFDM and incubated at room temperature for 1 hour. After incubation with secondary antibodies, membranes were washed 5 times for 5 minutes with TBST. Protein bands were visualized by exposing membrane after addition of ECL Western Blotting Substrate (Pierce) to X-ray film (Thermofisher Scientific) via standard procedures.

### RNA Extraction and Quantitative Real Time PCR (Q-RT-PCR)

RNA extraction was performed using RNeasy (Qiagen, Valencia CA) as per manufacturer protocol. Q-RT-PCR was performed using QuantaStudio7 Real-Time PCR System (Applied Biosystems) using a 96 well format. Reactions consisted of 5 ul of cDNA per reaction, 10 μl of 2 × Taqman Gene Expression Master Mix (Applied Biosystems) and 1 μl of 20 × Taqman probe, as well as nuclease-free water (total reaction volume 20 μl/well). The following Taqman probes with corresponding catalogue number for human genes were used in this study. PPARɣ (Hs00234592_m1), FOXA1 (Hs04187555_m1), GATA3 (Hs00231122_m1), KRT20 (Hs00300643_m1), KRT6A (Hs01699178_g1), UPK3A (Hs00199590_m1), EGFR (Hs01076078_m1). Relative gene expression was analyzed using deltadeltaCt method using 18 S ribosomal RNA as a reference. Significant differences in gene expression following experimental manipulation were identified by one-way ANOVA.

### Gene expression analysis by RNA-seq

Gene expression in modified cell lines was determined by RNA-seq. UC cell lines classified as neither luminal nor basal (“non-type”; see results section) were excluded from this portion of the analysis. For RNA quality control prior to RNAseq, optical density values of extracted RNA were measured using NanoDrop (Thermo Scientific) to confirm an A_260_:A_280_ ratio above 1.9. RNA integrity number (RIN) was measured using BioAnalyzer (Agilent) RNA 6000 Nano Kit to confirm RIN above 6. For RNAseq, SureSelect Strand Specific RNA Library Preparation Kit (Agilent) was used to generate cDNA libraries as per the manufacturer’s instructions. Briefly, polyA RNA was purified from 2 μg of total RNA using oligo (dT) beads. Extracted RNA underwent fragmentation, reverse transcription, end repair, 3′-end adenylation, adaptor ligation and subsequent PCR amplification and SPRI bead purification (Beckman Coulter). The unique barcode sequences were incorporated in the adaptors for multiplexed high-throughput sequencing. The final product was assessed for its size distribution and concentration using BioAnalyzer High Sensitivity DNA Kit (Agilent) and Kapa Library Quantification Kit (Kapa Biosystems). The libraries were pooled and diluted to 2 nM in EB buffer (Qiagen) and then denatured using the Illumina protocol. The denatured libraries were diluted to 10 pM by pre-chilled hybridization buffer and loaded onto TruSeq SR v3 flow cells on an Illumina HiSeq 2500 (Illumina) and run for 50 cycles using a single-read recipe (TruSeq SBS Kit v3, Illumina) according to the manufacturer’s instructions. For data analysis of RNAseq, Illumina CASAVA pipeline (released version 1.8, Illumina) was used to obtain de-multiplexed sequencing reads (fastq files). A bowtie2 index was built for the GRCh38 genome assembly using bowtie version 2.0.1. The RNA-seq reads of each sample were mapped using Tophat version 2.0.9^34^ supplied by Ensembl annotation file; GRCh38.78.gtf. Mapped reads were then used to quantify gene expression using Cufflinks^35^ version 2.2.1 supplied by GRCh38.78.gtf. Normalization was performed via the median of the geometric means for fragment counts across all libraries, as described by Anders and Huber^36^. Differences in gene expression patterns following the overexpression of FOXA1 and/or GATA3 in the presence and absence of rosiglitazone (all transfections and drug treatments were performed in duplicate for RNA-seq for a total of 16 samples) were determined by Classification of Nearest Centroid analysis and hierarchical clustering, and by directly comparing experimentally manipulated cell lines to luminal and basal cell lines identified in our analysis of CCLE data (see supplemental methods). In addition, the limma package^38^ was used to identify genes that were differentially expressed between treated and control cell lines. Because of the small sample size (n = 2 in both the treated and control groups), differentially expressed genes were identified based on fold change, not a statistical measure such as p- or q-value. Differential expression of transcription factors between luminal and basal cancers was determined using linear models (see supplemental methods).

### Chromatin immunoprecipitation and massively parallel DNA sequencing (ChIP-Seq)

For identification of FOXA1 bound elements by ChIP-Seq, RT4 cells were cultured as described above, and crosslinked with 1% paraformaldehyde (Sigma). Following extraction of DNA/protein complexes, samples were incubated with anti-FOXA1 antibody (Abcam; ab5089). Sequencing libraries were generated using Rubicon ThruPLEX DNA sequencing kit (Rubicon Genomics, Ann Arbor MI). Sequencing was performed on an Illumina HiSeq2500 (Illumina, San Diego CA). For ChIP-Seq data analysis, raw FASTQ files were aligned to hg19 reference genome using bowtie2 (version 2.2.6)^39^. Sam files were converted to bam using samtools (version 1.3)^40^ and peaks were called using HOMER (version 4.8)^41^. The two replicates were analyzed using Diffbind (version 2.05)^42^ R package where correlation coefficient and common peaks were extracted for further downstream analysis. De novo motif analysis for known transcription factors (TF) were generated with HOMER based on positional weight matrices from TRANSFAC (version 8.3). False-discovery rate of 5% was used as a cut-off.

## Results

### Analysis of publically available data through the CCLE identifies human cell lines suitable for the study of specific molecular subtypes of bladder cancer

**Figure 1:**
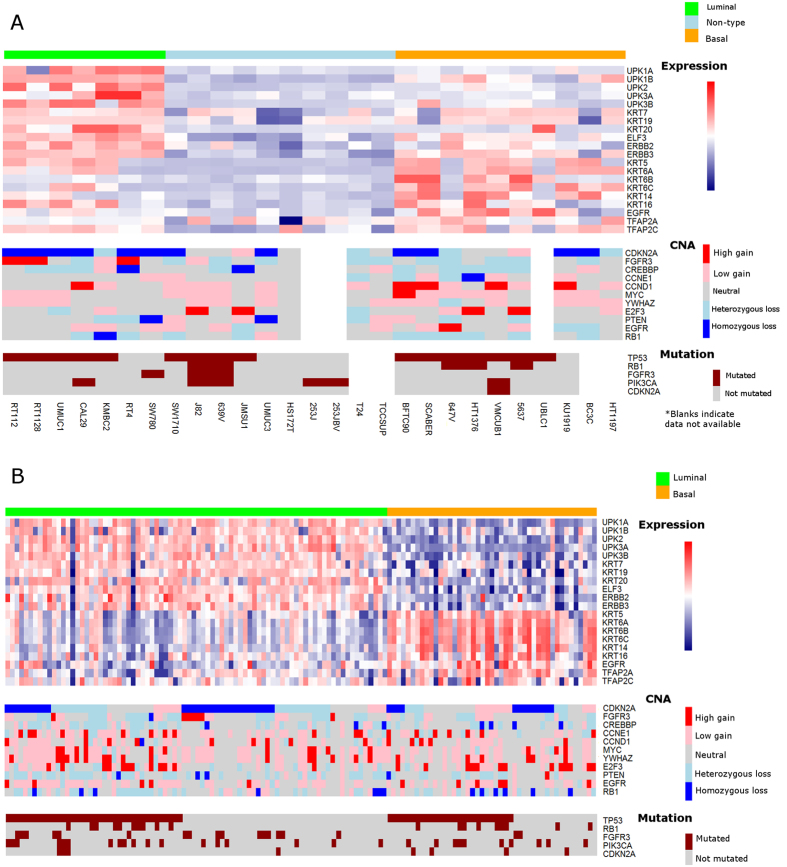
Comparison of CCLE gene expression and genetic data with gene expression and genetic data from the bladder TCGA study identifies human cell lines representative of luminal and basal bladder cancer. (**A**) CCLE Urinary Tract Cell Line Data, cases organized by expression subtype (Luminal, ”non-type”, and Basal), copy number alterations (CNA), and mutational data, from several genes commonly affected in human bladder cancer. (**B**) TCGA Urothelial Bladder Cancer Data, cases organized by expression subtype (luminal vs basal), copy number alterations (CNA), and mutational data from several commonly affected genes.

Recent studies have identified discrete molecular subtypes of UC^2^,^3^,^4^,^5^,^6^,^7^, with some reports indicating that these discrete subtypes respond differentially to multimodal therapy^8^,^43^, or even undergo transition to alternate molecular subtypes following treatment^2^. In order to evaluate the suitability of commonly used bladder cancer cell lines for the study of molecular subtypes of disease, as well as to identify specific mechanistic drivers of molecular subtype, we undertook a detailed analysis of gene expression, copy number alteration (CNA), and mutational data on 27 UC cell lines available through the CCLE^22^. Agglomerative methods performed on gene expression data, using the expression subtype gene list from the TCGA study, revealed UC cells lines formed three gene expression clusters (Fig. 1A): luminal (RT112, RT1128, UMUC1, CAL29, KMBC2, SW780 and RT4), basal (BFTC905, SCaBER, 647 V, HT1376, VMCUB1, 5637, UBLC1, KU1919, BC3C, and HT1197), and a group of cell lines showing low expression of luminal and basal markers, designated by us as “non-type” (SW1710, 639 V, J82, JMSU1, UMUC3, HS172T, 253 J, 253JBV, T24, and TCCSUP). In addition, mutations and CNAs associated with UC cell lines classified as luminal and basal mirrored those seen in the TCGA data (Fig. 1B). For example, *FGFR3* mutation and copy number gains were identified in luminal but not basal cell lines. In addition, *CDKN2A* copy number losses and *TP53* mutations were common in both luminal and basal cell lines, similar to human bladder cancers. Furthermore, centroids were constructed to define luminal and basal subtypes for TCGA and CCLE data, with genes chosen from analysis of CCLE data (see below and supplemental methods^44^). Good correlation was seen for luminal-TCGA vs luminal-CCLE centroids (r = 0.43; p = 0.0021, Spearman) and basal-TCGA vs basal-CCLE centroids (r = 0.29; p = 0.038, Spearman). Centroid-defining genes showed good separation of luminal from basal tumors in the CCLE and TCGA data sets using agglomerative methods (Figure S1). In conjunction with previous reports^23^, these observations identify a subset of UC cell lines potentially suitable for studies focused on understanding molecular subtypes of bladder cancer.

### Specific urothelial cancer morphologies correlate with molecular subtype assignment

Molecular subtype has been repeatedly correlated with tissue morphology in bladder cancer^3^,^4^,^43^, and variant histologies in bladder cancer are often associated with differences in clinical outcome^45^,^46^, much as is the case with molecular subtype^2^,^7^. Specifically, luminal bladder cancers are enriched for papillary histology in the noninvasive component, whereas basal bladder cancers are enriched for squamous differentiation^4^. In order to determine if a subset of UC cell lines classified as luminal or basal additionally exhibited a histomorphologic pattern consistent with molecular subtype assignment, we performed tissue recombination xenografting (see materials and methods) using RT4 (luminal), UMUC1 (luminal), and SCaBER (basal) UC cells.

**Figure 2:**
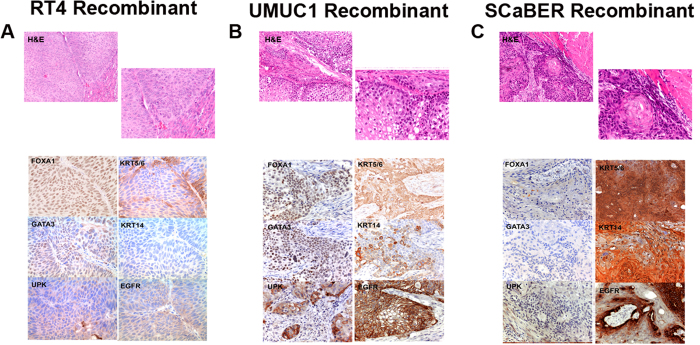
*In vivo* experiments show a subset of human cancer cell lines exhibits histomorphology and marker expression consistent with molecular subtype assignment. Hematoxylin and eosin stain (top panels) and immunohistochemistry for the luminal markers FOXA1, GATA3, and UPK, as well as the basal markers KRT5/6, KRT14, and EGFR following tissue recombination of (**A**) RT4, (**B**) UMUC1, and (**C**) SCaBER human bladder cancer cell lines.

As we previously reported, tissue recombinants containing RT4 cells (luminal) exhibited a morphologic pattern consistent with a high grade, non-invasive urothelial cell carcinoma, with papillary histology characterized by fibro vascular cores and no destructive invasion into the renal parenchyma (Fig. 2A, top panel). Immunohistochemical analysis of RT4-based tissue recombinants show that these tumors express FOXA1, GATA3, and uroplakins, as well as the basal markers KRT5/6, but fail to express the basal markers KRT14 and EGFR (Fig. 2A; bottom panels). Tissue recombinants containing UMUC1 (luminal) had a conventional invasive urothelial carcinoma morphology (Fig. 2B, top panel). Interestingly, UMUC1 cells were extremely aggressive, and exhibited deep invasion into the kidney (Fig. 2B H&E) as well as local extension into adjacent structures. Immunohistochemical analysis of UMUC1-based recombinants revealed these tumors expressed a subset of both luminal and basal markers (Fig. 2B; bottom panels) consistent with our gene expression analysis (Fig. 1). This observation, as well as additional immunohistochemistry for KRT20 and specific uroplakin family members (see supplementary Figure 4) suggests a limited panel of markers is not sufficient to differentiate a relatively small set of xenograft tumors as belonging to a specific molecular subtype. Tissue recombinants consisting of SCaBER cells (basal), a cell line established from a patient with pure squamous cell carcinoma^29^, exhibited an invasive phenotype with intercellular bridges and keratin pearls, both hallmarks of squamous differentiation (Fig. 2C, top panel). SCaBER tissue recombinants failed to express detectable levels of FOXA1, GATA3, and UPK, but did express high levels of KRT5/6, KRT14, EGFR (Fig. 2C, bottom panels) and UPK2 (supplemental Figure S4). Taken together, pathological characterization of *in vivo* tumors derived from RT4, UMUC1, and SCaBER cells support our gene expression and genetic analysis of these cell lines. These results further support the use of these cell lines as models of high-grade non-invasive luminal bladder cancer (RT4), high-grade invasive luminal bladder cancer (UMUC1), and high-grade invasive basal bladder cancer with squamous differentiation (SCaBER).

### Molecular subtype of human bladder cancer cell lines is associated with the differential expression of transcription factors in a manner similar to human bladder cancer samples

**Figure 3:**
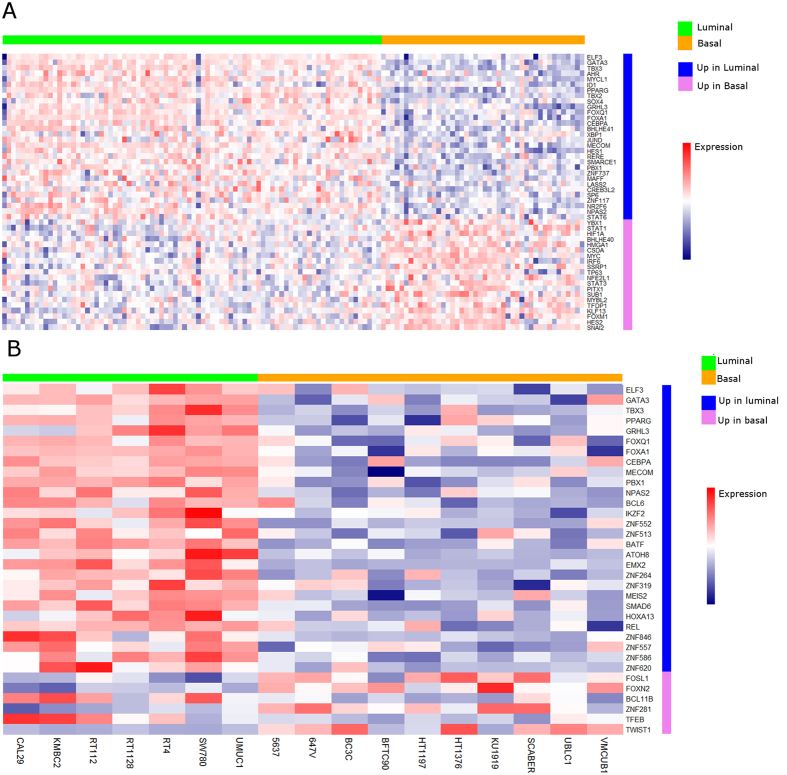
Comparison of bladder TCGA and CCLE urothelial cancer cell line data identifies parallels in differential expression of transcription factors in human cancers and bladder cancer cell lines, respectively. Transcription factors differentially expressed between luminal and basal human bladder cancers in TCGA data and CCLE data sets. (**A**) TCGA data set - top 50 differentially expressed genes (q < 0.1, t-statistic from linear models; all fold-change > 1,000). (**B**) CCLE data set – 34 genes differentially expressed between luminal and basal cell lines (q < 0.1, t-statistic from linear models; genes drawn from list of genes differentially expressed in TCGA data set).

Based on our gene expression and *in vivo* phenotypic analysis of human bladder cancer cell lines relative to human clinical samples, we next determined which transcription factor expression networks were linked to specific molecular subtypes. We analyzed the expression levels of ~1,500 transcription factors^47^ in luminal and basal tumors from the TCGA data set (see supplemental methods). Of these, we identified 468 transcription factors as being overexpressed in luminal cancers and 194 transcription factors as being overexpressed in basal cancers (q < 0.1, t-statistic from linear model^38^; Fig. 3A). Of these 662 differentially expressed transcription factors, 32 were differentially expressed between luminal and basal cell lines in the CCLE data set, including several markers of urothelial differentiation such as GATA3^14^, ELF3^14^,^20^, GRHL3^15^, FOXA1^9^, and PPARɣ^11^ (q < 0.1, t-statistic from linear model, Fig. 3B). In summary, a subset of established human UC cell lines exhibit a transcription factor expression pattern similar to human bladder cancer samples, providing further evidence of the suitability of these cell lines for studies focused on molecular subtype in bladder cancer.

### GATA3, FOXA1, and PPARɣ cooperate to regulate a subset of luminal and basal markers of bladder cancer

**Figure 4:**
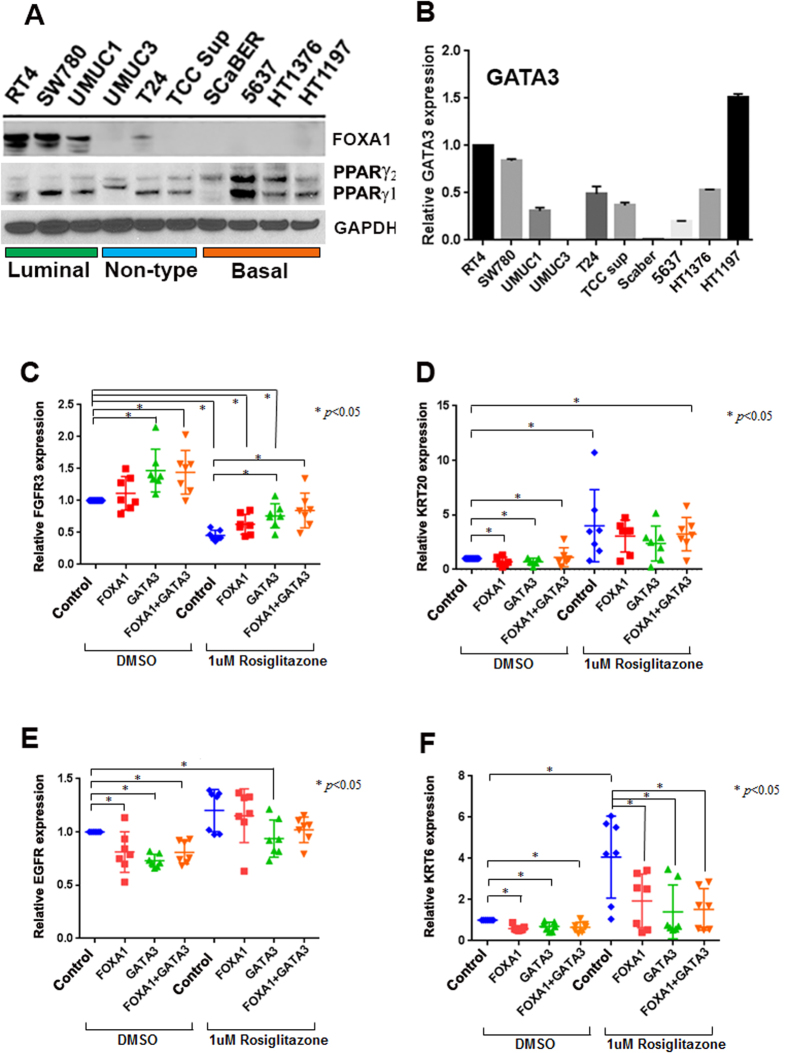
Candidate gene analysis suggests GATA3 and FOXA1 cooperate with PPARɣ activation to regulate molecular subtype-specific gene expression in bladder cancer cells. (**A**) Western blotting analysis of a panel of human bladder cancer cell lines for expression of FOXA1 and PPARɣ. Cells are arranged by molecular subtype based on analysis presented in Fig. 1. (**B**) Q-RT-PCR analysis of human bladder cancer cell lines for GATA3. (**C–F**) Q-RT-PCR analysis for the luminal bladder cancer markers (**C**) FGFR3 and (**D**) KRT20, as well as the basal bladder cancer markers (**E**) EGFR and (**F**) KRT6 following individual overexpression of empty vector (CMV), FOXA1, GATA3, or FOXA1 and GATA3 in the presence of vehicle control (DMSO) or 1 micromolar rosiglitazone (TZD). See materials and methods for detailed description of experimental approach.

Studies have shown that PPARɣ plays an important role in regulating the expression of genes associated with the luminal molecular subtype of muscle invasive bladder cancer^2^,^21^. In addition, FOXA1 expression is required for the maintenance of urothelial differentiation^9^, and alterations in FOXA1 are common in bladder cancer^4^,^18^. While FOXA1 is reportedly a PPARɣ target gene^48^, it is unknown if these transcription factors cooperate to regulate genes associated with molecular subtypes of bladder cancer. Moreover, while GATA3 is differentially expressed in histologic variants of UC^49^, and overexpression of GATA3 is associated with the luminal molecular subtype^4^,^12^, it is unknown how GATA3 regulates the expression of genes associated with molecular subtypes of bladder cancer. Based on our analysis of differentially expressed transcription factors identified through the TCGA bladder study, and our characterization of bladder cancer cell lines showing similar transcription factor expression patterns (Fig. 3), we chose to examine the impact of GATA3 and FOXA1 expression on the regulation of markers of luminal and basal bladder cancer in the presence and absence of the PPARɣ agonist rosiglitazone. By western blot analysis of FOXA1 in a subset of bladder cancer cell lines included in the CCLE data and cultured in our laboratory, we found that other than a small amount of FOXA1 detected in the “non-type” T24 cells, FOXA1 expression was restricted to the luminal bladder cancer cell lines (Fig. 4A). Western blotting analysis for PPARɣ showed that this receptor was detected to various degrees in all of the cell lines. However, we noted that the luminal cell lines largely expressed PPARγ1, while 3 out of 4 basal cell lines expressed both PPARγ1 and PPARγ2 (Fig. 4A). Similarly, while GATA3 transcript was detected to various degrees in the luminal cell lines, the “non-type” cell lines and the basal cell lines also expressed GATA3 (Fig. 4B). Based on the observation that the cell line 5637 exhibits undetectable levels of FOXA1 and low levels of GATA3, but expresses high levels of both PPARɣ isoforms, we chose this basal cell line for additional studies. Specifically, we sought to determine the impact of transient overexpression of GATA3 and FOXA1, individually and in combination with each other, and in both the absence and presence of the PPARɣ agonist rosiglitazone, on the expression of markers of luminal (FGFR3 and KRT20) and basal (EGFR and KRT6) bladder cancer. Interestingly, GATA3 overexpression alone was sufficient to increase expression of FGFR3 in 5637 cells (Fig. 4C), whereas rosiglitazone treatment repressed FGFR3 expression, which was partially rescued by GATA3 alone and/or in combination with FOXA1 (Fig. 4C). On the other hand, KRT20 expression was only marginally (albeit significantly) increased by the overexpression of GATA3 and FOXA1 alone or in combination with each other, whereas rosiglitazone treatment alone was sufficient to dramatically increase expression of KRT20 (Fig. 4D). Expression of EGFR was significantly repressed by overexpression of GATA3 or FOXA1, alone and in combination, while rosiglitazone treatment increased EGFR expression (Fig. 4E), as was the case for KRT6 (Fig. 4F). We characterize 5637 cells as representative of the basal molecular subtype of human disease (Fig. 1). In order to determine the impact of FOXA1 and/or GATA3 overexpression on the expression of basal markers at the protein level in this cell line, we performed western blotting for KRT5/6 and EGFR (see supplemental figure S5). Western blotting analysis revealed that overexpression of FOXA1 and/or GATA3 had no effect on KRT5/6 expression, even in the presence of rosiglitazone (supplemental figure S5). However, western blotting revealed that GATA3 overexpression appeared repress EGFR expression, and this was enhanced following rosiglitazone treatment (supplemental figure S5). This is interesting, as the GATA3 paralog GATA6 was shown to be a negative regulator of EGFR^50^. In summary, the impact of individual and combined GATA3 and FOXA1 expression, as well as PPARɣ activation, results in statistically significant, marker-dependent changes in gene expression at the transcriptional level in 5637 cells. These results suggested to us that a less biased and more broadly-based approach for data analysis was required, which takes into account expression of a much larger number of genes.

### Multiple gene-based analysis identifies a coordinated transcription factor network consisting of GATA3, FOXA1, and PPARɣ that activates a luminal gene expression program in bladder cancer cells

**Figure 5:**
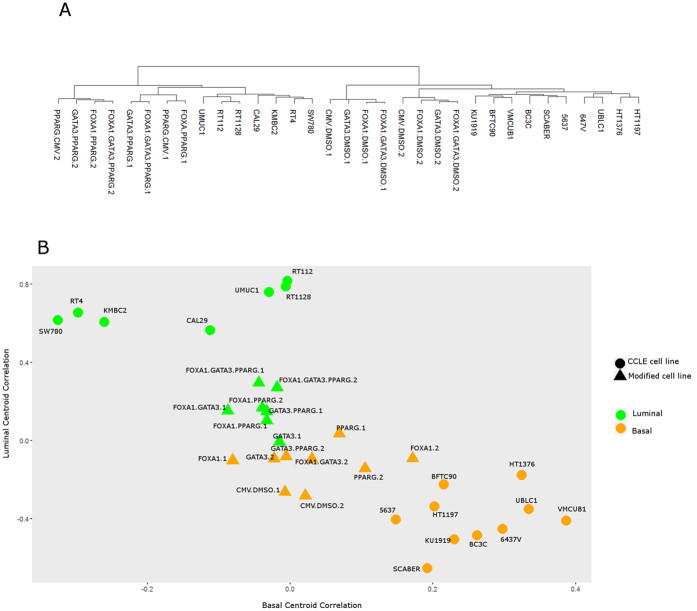
Analysis of RNA-seq data identifies GATA3 and FOXA1 as cooperating with PPARɣ to promote luminal gene expression in bladder cancer cells. (**A**) Hierarchical clustering of CCLE urinary tract cell lines and 5637 (basal) cell lines following overexpression of GATA3 and/or FOXA1 in the presence and absence of 1 micromolar rosiglitazone (PPARɣ agonist). Cell lines are divided into two large clusters, one containing CCLE Basal cell lines and 5637 cell lines modified by FOXA1 and/or GATA3, and another containing CCLE Luminal cell lines and 5637 cell lines modified by addition of multiple factors or rosiglitazone treatment alone. (**B**) Nearest centroid analysis shows modified cell lines move closer to the luminal centroid with addition of multiple factors, with FOXA1.GATA3.PPARG cell lines being closest to the luminal centroid. No single factor was able move 5637 sufficiently close to the luminal centroid to classify the cell lines as luminal.

In an effort to perform an unbiased analysis regarding the impact of altered GATA3 and FOXA1 expression and PPARɣ activity on molecular subtype, as well as the extent to which these factors cooperated to regulate global gene expression, we performed RNA-Seq analysis on 5637 cells transiently overexpressing GATA3 and/or FOXA1 in the presence and absence of the PPARɣ agonist, rosiglitazone. Using a fold change of 2 as the threshold for identifying differentially expressed genes, n = 310 protein coding genes were upregulated in rosiglitazone treated 5637 cells overexpressing FOXA1 and GATA3 when compared 5637 cells transfected with empty vector and treated in the presence of DMSO, while n = 730 protein coding genes were downregulated (see supplemental figure S6). Hierarchical clustering of our RNA-seq data with expression data from CCLE luminal and basal cell lines placed our 5637 control cells in a cluster with CCLE basal cell lines (Fig. 5A), consistent with a previous study^43^.Rosiglitazone treatment resulted in the placement of 5637 cell lines with activated PPARɣ signaling in the same cluster as luminal CCLE cell lines (Fig. 5A), also consistent with previous studies indicating an important role for PPARɣ^2^,^21^ On the other hand, individual or combined expression of GATA3 and FOXA1 was incapable of resulting in the re-clusting of 5637 gene expression data into the luminal category (Fig. 5A). However, we were still unable to determine the extent to which (if any) PPARɣ, GATA3, and FOXA1 cooperate to establish a luminal molecular subtype in basal 5637 cells. Therefore, Classification of Nearest Centroid analysis (ClaNC) was used to more precisely investigate the impact of combinatorial expression of GATA3, FOXA1 and PPARɣ on development of a luminal expression signature (Fig. 5B). A nearest centroid predictor, which uses 50 genes optimally chosen from the TCGA expression subtype gene list (see supplemental protocols) was used to assign all experimentally manipulated 5637 cells as belonging to the luminal or basal expression subtype^44^. As expected, 5637 cells transiently transfected with empty vector plasmid and treated with vehicle control were classified as basal by this method (Fig. 5B). Movement of 5637 cells toward the luminal centroid was seen with the addition of GATA3 alone, FOXA1 alone, and following PPARɣ activation alone. However, no single factor, including PPARɣ activation in 5637 cells by rosiglitazone, conferred sufficient movement to classify experimentally manipulated cells as luminal in both replicates (Fig. 5B). In contrast, both replicates of 5637 cells treated with PPARɣ agonist and overexpressing FOXA1, and both replicates with PPARɣ and combined GATA3 and FOXA1, exhibited sufficient movement toward the luminal centroid to classify as luminal (Fig. 5B). No cell line without PPARɣ activation was classified as luminal in both replicates. Based on ClaNC analysis, our results show that individual overexpression of GATA3 or FOXA1, or activation of PPARɣ alone in 5637 cells, is insufficient to drive a gene expression pattern that qualified as luminal. While our conclusions are limited to this one cell line, our analysis indicates these factors act in concert and cooperate with PPARɣ to reprogram the basal bladder cancer cell line 5637, resulting in activation of a gene expression pattern consistent with the emergence of a luminal molecular subtype.

### Identification of FOXA1 occupied *cis* regulatory regions in RT4 cells reveals a complex network of potential transcriptional co-regulators

**Figure 6:**
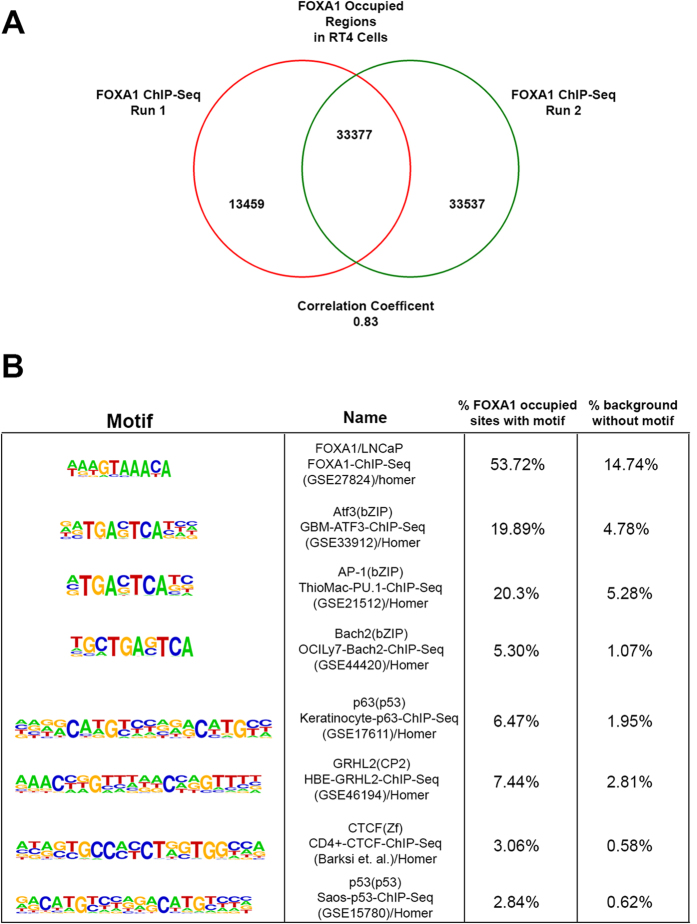
ChIP-seq for FOXA1 occupied regions in luminal RT4 bladder cancer cells identifies novel transcription factors that potentially cooperate with FOXA1 to regulate gene expression. (**A**) Venn diagram showing the number of FOXA1 binding sites identified by replicate ChIP-seq experiments to be highly concordant. (**B**) Subset of transcription factor binding sites identified by motif analysis as being associated with FOXA1 occupied regions in RT4 cells (see supplemental data for complete list).

In order to obtain information regarding the extent to which FOXA1 cooperates with GATA3 and PPARɣ, as well as to identify other transcription factors that potentially regulate gene expression in a luminal bladder cancer cells, we performed chromatin immunoprecipitation of FOXA1 in RT4 cells followed by high throughput next generation sequencing (ChIP-Seq). This approach resulted in the identification of 33,377 shared FOXA1 binding sites following two separate ChIP-Seq experiments (Fig. 6A). In addition, motif analysis identified 172 motifs significantly associated with FOXA1 occupied sites (q < 0.05; see supplemental table ST1), several of which were unanticipated. For example, binding sites for the stress response transcription factor ATF3 were the most enriched over background (Fig. 6B, not including FOXA1 and other forkhead factors). In addition, binding sites for AP-1 were enriched near FOXA1 occupied regions (20.3% of FOXA1 occupied sites associated with an AP-1 motif, compared to 5.28% of background; Fig. 6B). The association of AP-1 bound sites was interesting, as binding sites for several components of the AP-1 pathway (FRA1, BATF, FOSL2, JUN-AP1) were frequently enriched near FOXA1 occupied regions (see supplementary table ST1). In addition, binding sites for TP63 (6.47% of FOXA1 occupied regions versus 1.95% of background) and TP53 (6.47% of FOXA1 occupied regions versus 1.95% of background) were also associated with FOXA1 occupied regions relative to background (Fig. 6B). However, we were surprised that motif analysis identified a minimal amount of GATA3 binding sites (q < 0.05; 2.91% of FOXA1 occupied regions versus 1.11% of background) and PPARɣ binding sites (q < 0.05; 7.86% of FOXA1 occupied regions versus 5.79% of background) as being associated with FOXA1 occupied regions. This observation suggested to us that additional transcriptional regulators identified by this process almost certainly play a role in cooperating with FOXA1 to regulate gene expression in luminal RT4 cells. However, as overexpression of FOXA1 combined with PPARɣ activation was sufficient to promote a luminal gene expression pattern (Fig. 5B), we performed additional analysis of our ChIP-Seq data to identify biological processes and pathways, with a focus on FOXA1 occupied regulatory elements associated with GATA3 and PPARɣ binding sites identified by motif analysis. Molecular Signature Database (MSigDB) pathway misspelled identified the platelet-derived growth factor receptor (PDGFR alpha) signaling pathway as being associated with genes as potentially co-regulated by FOXA1, GATA3 and PPARɣ (see supplemental Figure S7). In addition, MSigDB Perturbation analysis of FOXA1 occupied regions with associated GATA3 and PPARɣ motifs identified a number of gene sets expression signatures of interest, including a set of genes with associated p53 binding sites (see supplemental Figure S8), and this observation supported our motif analysis. In summary, our ChIP-Seq experiments identify AP-1 factors, as well as additional unanticipated factors as potentially cooperating with FOXA1 to co-regulate genes in RT4 cells.

### Coordinated analysis of human and cell line expression data identifies a set of regulated transcription factors potentially contributing to the emergence of a luminal molecular subtype

**Figure 7:**
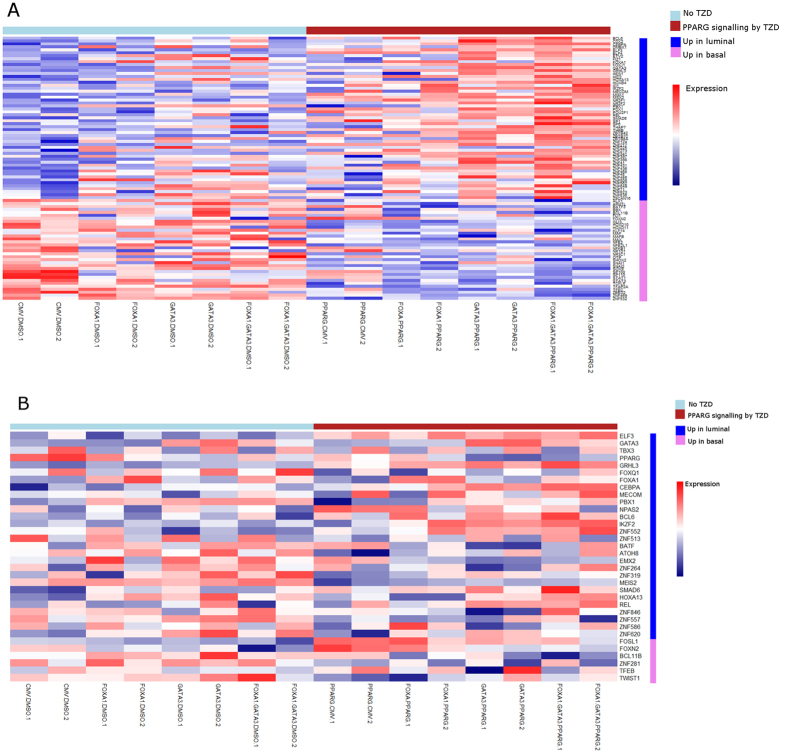
GATA3, FOXA1, and PPARɣ alter the expression of transcription factor networks in human bladder cancer cells. Transcriptions factors upregulated and downregulated by addition of GATA3 and/or FOXA1 in the presence or absence of PPARɣ agonist treatment (TZD). (**A**) Transcription factors with largest fold-change between our control 5637 cell lines and FOXA1.GATA3.PPARG modified cell lines. (**B**) Transcription factors identified as differentially expressed between luminal and basal cell lines in the CCLE data set (Fig. 3B), applied to our modified cells lines.

While our experimental data indicated GATA3 and FOXA1 cooperate with PPARɣ activation to drive basal to luminal transdifferentiation in 5637 cells, we were curious if the regulation of additional transcription factors associated with molecular subtypes of UC played a role in our experimental system. Therefore, we used gene expression data following the individual and combined overexpression of GATA3 and FOXA1 in the presence and absence of PPARɣ agonist to identify additional transcriptional regulators that may play a role in the activation of a luminal bladder cancer-specific gene expression. Strikingly, overexpression of GATA3 and FOXA1 in the presence of PPARɣ agonist modulated the expression of several transcription factors that are differentially expressed between luminal and basal human bladder cancers, including ELF3, SNAI1, and SNAI2 (both implicated in epithelial to mesenchymal transition^51^) and TFAP2A^52^ among others (Fig. 7, see supplemental protocol). This was also seen to a lesser degree in other modified cell lines. These results suggest alterations leading to the combined overexpression of GATA3 and FOXA1, and enhanced activation of PPARɣ signaling, cooperate with changes in the expression of other transcription factors to regulate the switch from a basal to luminal molecular subtype in basal 5637 cells. Our data also indicates this occurs by altering the expression of key downstream transcription factors implicated in bladder cancer.

## Discussion

Clinical outcomes for the treatment of muscle invasive bladder cancer are enhanced following use of multimodal therapy, which includes neoadjuvant, cisplatin-based chemotherapy followed by cystectomy^53^. Indeed, downstage to pT0 following neoadjuvant chemotherapy is significant positive indicator, suggesting primary bladder tumors are representative surrogates of how circulating and disseminated tumor cells, as well as established metastatic lesions, respond to systemic chemotherapy. However, 50% of patients with muscle invasive bladder cancer succumb to disease following multimodal therapy, and it is currently impossible to identify which patients will respond to neoadjuvant chemotherapy. In addition, while use of immunotherapy following the failure of first-line multimodal approaches is enhancing survival in patients^54^, clinical response is generally poor in patients with recurrent/progressive bladder cancer following initial treatment. Therefore, increased effort is needed to identify better treatments for these patients.

There can be no doubt that limited improvements in the clinical management of bladder cancer in recent decades are a direct reflection of our lack of knowledge in the cell and molecular biology of the urothelium. However, recent molecular characterization studies, including the molecular data provided by the TCGA bladder effort, provide an outstanding opportunity to identify novel mechanisms responsible for the malignant phenotype, as well as actionable genetic alterations associated with aggressive disease. Importantly, this unique opportunity to improve the clinical management of disease requires robust experimental systems representative of human disease which can be used for translational research. For this reason, we have capitalized on molecular data available through the TCGA, to undertake a comprehensive analysis of 27 human bladder cancer cell lines available through the CCLE. Through this approach we identified a subset of cell lines that are representative of luminal and basal molecular subtypes of bladder cancer. In regard to the assignment of specific cell lines as basal, our results are largely in agreement with Rebouissou *et. al.*^43^ and Earl *et. al.*^23^. In the current study, we have now additionally identified cell lines suitable for experiments focused on elucidating cancer biology associated with the luminal molecular subtype of bladder cancer. Importantly, the molecular subtypes represented in these cell lines are also associated with genetic alterations typically associated with a given subtype in human tumors as identified by TCGA. For example, luminal bladder cancer cell lines were enriched for mutation and amplifications in *FGFR3*, while mutations in *TP53* and deletions of *CDKN2A* were more evenly distributed throughout the cell lines examined, much as is the case with clinical disease. In addition, our analysis of tumor tissue collected following *in vivo* tissue recombination xenografting experiments show that a subset of cell lines exhibit histology consistent with their molecular subtype assignment, with luminal cell lines exhibiting a non-invasive papillary or conventional invasive UC phenotype, whereas the tested basal cell line exhibited an invasive phenotype with squamous differentiation. In short, our analysis, and the analysis of other groups^23^,^43^ has helped to establish the relevance of these cell lines for the design of experiments to understand the role of molecular subtype in bladder cancer.

One of the most striking observations made during our comparisons of gene expression in the CCLE cell lines and human tumors was the degree to which key transcriptional regulators were correlated with a luminal and basal gene expression signature in both data sets. Similar to what other groups have observed^12^, this observation suggested a predominant role of transcriptional regulators in the regulation of molecular subtype. However, the level of evidence regarding the role of specific transcription factors in the establishment/regulation of a given molecular subtype varies. For instance, previous experimental studies indicate an important role for PPARɣ and EGFR in the positive regulation of genes associated with the luminal and basal molecular subtypes of bladder cancer, respectively^2^,^21^,^43^. Similarly,FOXA1 was recently identified as a target of microRNA-99a and −100^55^, both of which are diminished in a subset of FOXA1 and FGFR3 overexpressing luminal bladder cancers^4^. Our previous xenografting and inducible knockout studies^9^,^18^ have also established a role for FOXA1 in bladder cancer, and suggest a direct contribution of this factor to the luminal subtype. In addition, while recent analysis of publically available gene expression and ChIP-seq data is strongly suggestive of a direct role for GATA3 and other novel factors in the luminal molecular subtype of bladder cancer^12^, to our knowledge there is no direct evidence regarding a role of GATA3 in the establishment of molecular subtype. In addition, the extent to which these factors cooperate with each other to establish and/or maintain molecular subtype is unknown. Accordingly, our integrated analysis of CCLE and TCGA data provided us a unique opportunity to design experiments to directly test the individual and combined contribution of GATA3, FOXA1, and PPARɣ to the regulation of genes associated with the luminal molecular subtype of bladder cancer, as well as the establishment of the luminal molecular signature in bladder cancer cells. Because of the complex nature of this data, we chose a detailed approach incorporating both hierarchical clustering and Classification of Nearest Centroid (ClaNC) analysis, and we feel this is a particularly innovative approach for the analysis of experimental data. Significantly, we show that pharmacologic activation of PPARɣ is sufficient to cluster the basal cell line 5637 into a large cluster containing luminal cell lines, a finding consistent with a previous reports identifying an important role for PPARɣ in the activation of luminal-specific genes expressed in bladder cancer cells^2^,^21^. However, ClaNC analysis shows that 5637 cells cannot be classified as transitioning from basal to luminal by any single factor alone, including PPARɣ activation. Rather, our analysis shows that GATA3 and/or FOXA1 cooperate with PPARɣ activation to reprogram basal 5637 cells to a luminal molecular subtype. Our in-depth approach of data analysis understandably limits us to the analysis of one cell line following rosiglitazone treatment and overexpression of FOXA1 and GATA3. Therefore, while we must be cautious in how we interpret this data, our approach validates the importance of PPARɣ and additionally shows a direct impact of GATA3 and FOXA1 on the expression of markers of the luminal molecular subtype of bladder cancer. Most importantly, our data show that these factors can cooperate to establish a given molecular subtype. While it is important that future studies examine the effect of manipulating these factors in other bladder cancer cell lines, these findings have important implications for the potential “plasticity” of molecular subtype which reportedly can be altered following multimodal therapy^2^, or even possibly during tumor progression.

## Additional Information

**How to cite this article**: Warrick, J. I. *et al*. FOXA1, GATA3 and PPARɣ Cooperate to Drive Luminal Subtype in Bladder Cancer: A Molecular Analysis of Established Human Cell Lines. *Sci. Rep.* **6**, 38531; doi: 10.1038/srep38531 (2016).

**Publisher’s note:** Springer Nature remains neutral with regard to jurisdictional claims in published maps and institutional affiliations.

## Supplementary Material

Supplemental Methods and Data
