# Supplementary material for: FOXA1, GATA3 and PPARɣ Cooperate to Drive Luminal Subtype in Bladder Cancer: A Molecular Analysis of Established Human Cell Lines
Source: Sci Rep. 2016 Dec 7;6:38531. doi: 10.1038/srep38531 (PMC5141480; doi:10.1038/srep38531)

FOXA1, GATA3 and PPARɣ Cooperate to Drive Luminal Subtype in Bladder Cancer: A Molecular Analysis of Established Human Cell Lines

Joshua I. Warrick[1,2*], Vonn Walter[3,4], Hironobu Yamashita[1], Eunah Chung[5], Lauren Shuman[1,2], Vasty Osei Amponsa[1], Zongyu Zheng[1], Wilson Chan[1,2], Tiffany L. Whitcomb[5], Feng Yue[3,6], Tejaswi Iyyanki[3], Yuka I. Kawasawa[3,6], Matthew Kaag[2], Wansong Guo[7], Jay D. Raman[3], Joo-Seop Park[5], David J. DeGraff[1,2,3*]

[1]Department of Pathology, Pennsylvania State University Milton S. Hershey Medical Center, Hershey PA
[2]Department of Surgery, Division of Urology, Pennsylvania State University College of Medicine
[3]Department of Biochemistry and Molecular Biology, Pennsylvania State University College of Medicine
[4]Department of Public Health Sciences, Pennsylvania State University College of Medicine
[5]Division of Pediatric Urology and Developmental Biology, Cincinnati Children's Hospital Medical Center
[5]Department of Comparative Medicine, Pennsylvania State University College of Medicine
[6]Institute for Personalized Medicine, Pennsylvania State University College of Medicine
[7]Department of Surgery, Division of Urology, Changchun Central Hospital, Changchun, China
[*]Corresponding Authors

Supplemental protocols

TCGA and CCLE data comparison

For comparison between CCLE cell lines and human tumors, TCGA data from muscle invasive urothelial bladder cancer were obtained from the TCGA data portal (https://tcga-data.nci.nih.gov/tcga/). RNA-seq and mutational data were included. Cases with expression subtyping data were selected (n=127, https://tcga-data.nci.nih.gov/docs/publications/blca_2013).TCGA type 1 and 2 tumors were considered Luminal, and types 3 and 4 considered basal[15]. Similarly, CCLE expression data by microarray, mutational data (Oncomap), and SNP array data were included (http://www.broadinstitute.org/ccle/home).

Copy number alterations for TCGA data were taken directly from gene-level copy number calls from firebrowse.org (http://firebrowse.org/?cohort=BLCA&download_dialog=true). Based on these data, percentiles of discreet copy number alterations were determined, yielding: homozygous loss (-2), 0.69%; heterozygous loss (-1), 24%; copy number neutral (0), 51%; low-level copy number gain (1), 23%; high-level copy number gain (2), 2%. CCLE .seg files were used to determine copy number alterations in cell lines. Segmentation mean values falling into the approximate percentiles as the TCGA were utilized, yielding: homozygous loss (-2), segmentation mean=-0.4; heterozygous loss (-1), segmentation mean=-0.2; low-level copy number gain (1), segmentation mean=0.2; high-level copy number gain (2), segmentation mean=0.4. Veracity of this approach in the CCLE data was validated by comparing *CDKN2A* copy number loss assignment with CDKN2A

expression levels, which showed marked loss of expression in cases with homozygous *CDKN2A* copy number loss (p<0.001, Figure S2).

Expression clusters in CCLE data

Normalized microarray-derived expression data for urinary tract cell lines (n=27) were downloaded from the CCLE data portal (http://www.broadinstitute.org/ccle/data/browseData?conversationPropagation=beginc). The list of 2,708 gene used in the TCGA study was downloaded (https://tcga-data.nci.nih.gov/docs/publications/blca_2013/BLCA_gnames.txt), and the CCLE expression matrix was limited to these genes (2,556 genes from list in CCLE bladder cancer expression data). The expression matrix was log2 transformed. Agglomerative clustering was performed using correlation distance on the log2 transformed expression matrix using Ward's method, McQuitty's method, complete linkage, and average linkage to produce four dendrograms (figureS3). Ward's and McQuitty's methods placed cell lines in the same clusters when each was cut into three separate clusters (figure S2 green lines). Complete linkage and average linkage gave similar results, though these methods placed a small number of cell lines in their own smaller clusters. Expression subtype of cell lines was assigned as luminal, basal, or non-type based on the cluster assignments identified by Ward's and McQuitty's methods.

Cluster centroids were determined using cluster assignments described above, using luminal and basal cell CCLE lines only, based on log2 normalizeddata. The ClaNC package was utilized[34]. Cross-validation errors rates were minimal when including 25 genes per subtype (class), for a total of 50 genes used in centroid construction. A dendrogram (agglomerative clustering, average linkage, Euclidean distance) using these 50 genes showed clearly separated clusters (figure S1A). Correlation distance between each cell line (including our modified cell lines and the CCLE cell lines, both log2 normalized and scaled) and the luminal and basal centroids was determined. Modified cell lines were assigned to the subtype with the greatest correlation to the corresponding centroid vector (Figure 5).

A list of ~1,500 human transcription factors was obtained[37]. Differential expression of these transcription factors was determined between luminal (type I and II) and basal (types III and IV) cancers in the TCGA case set detailed above using the limma package in R[38], using TCGA data from the 127 cases detailed above. 468 transcription factors were identified as overexpressed in luminal tumors (q<0.1, t-statistic from linear model),

and 194 overexpressed in basal tumors (q<0.1, t-statistic from linear model). Using this selected set of 662 transcription factors, expression levels were compared between our control HTB9 cell lines and matched FOXA1.GATA3.PPARG cell lines. Specifically, transcription factors with fold-2 change in the expected direction (i.e. elevated in luminal genes and decreased in basal genes) were identified in 82 luminal genes and 34 basal transcription factor genes (Figure 6A).

Supplemental Figure Legends

ST1: Transcription factor motifs identified as being associated with FOXA1 occupied regions by ChIP-Seq in RT4 bladder cancer cells.

S1: Hierarchical clustering of Luminal and Basal cell lines using the 50 gene list used to define Luminal and Basal centroids, derived from the ClaNK package. This gene list robustly separates Luminal from Basal in both (A) CCLE data and (B) TCGA data.

S2: CDKN2A expression by *CDKN2A* copy number status in CCLE cases. Markedly low expression is present in cases with homozygous *CDKN2A* loss vs copy number neutral (p<0.001, Wilcoxon rank sum test).

S3: Hierarchical clustering dendrograms for CCLE data using multiple methods. Green lines indicate where dendrograms were cut for cluster assignment.

S4: Immunohistochemistry of RT4, UMUC1 and SCaBER tissue recombinants for KRT20 and members of the uroplakin family of proteins.

S5: KRT5/6 and EGFR western blotting of cell lysates extracted from 5637 bladder cancer cells transiently transfected with empty vector, FOXA1, GATA3 and FOXA1 and GATA3, and subsequently treated in the presence or absence of rosiglitazone. See materials and methods for description of experimental approach.

S6: Heatmap display of genes that are differentially expressed between treated and normal cell lines following the overexpression of FOXA1 and GATA3 in 5637 bladder cancer cells treated with rosiglitazone (n = 310 up in treatment, n = 730 down in treatment). Genes appear in rows and the expression values are median centered by gene.

S7: MSigDB Pathway analysis of FOXA1 ChIP-Seq data, with a focus on genes associated with FOXA1 occupied regions and associated GATA3 and PPARɣ binding sites identifies an association with the PDGFR-alpha signaling pathway.

S8: MSigDB Perturbation analysis of FOXA1 ChIP-Seq daa with a focus on genes associated with FOXA1 occupied regions and associated GATA3 and PPARɣ binding sites

S9: Analysis of FOXA1 ChIP-Seq data to identify distance of FOXA1 occupied regions with associated GATA3 and PPARɣ binding sites to the transcription start site (TSS) to a given gene.

Figure S1A

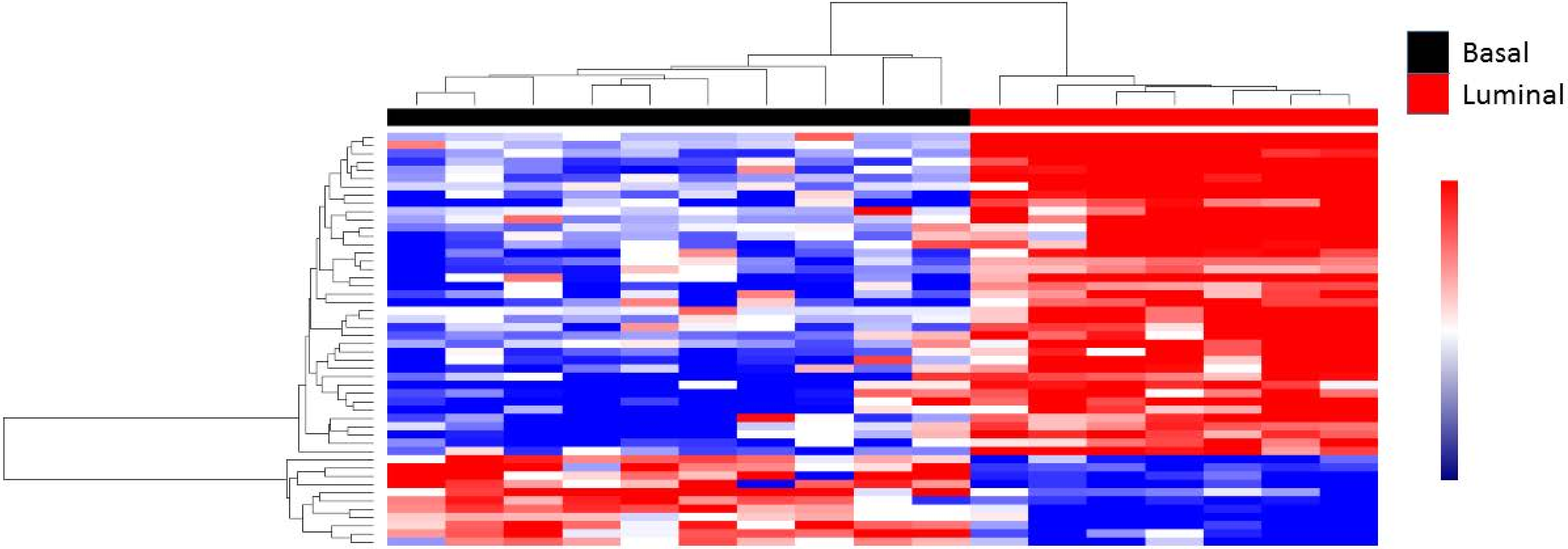

Figure S1B

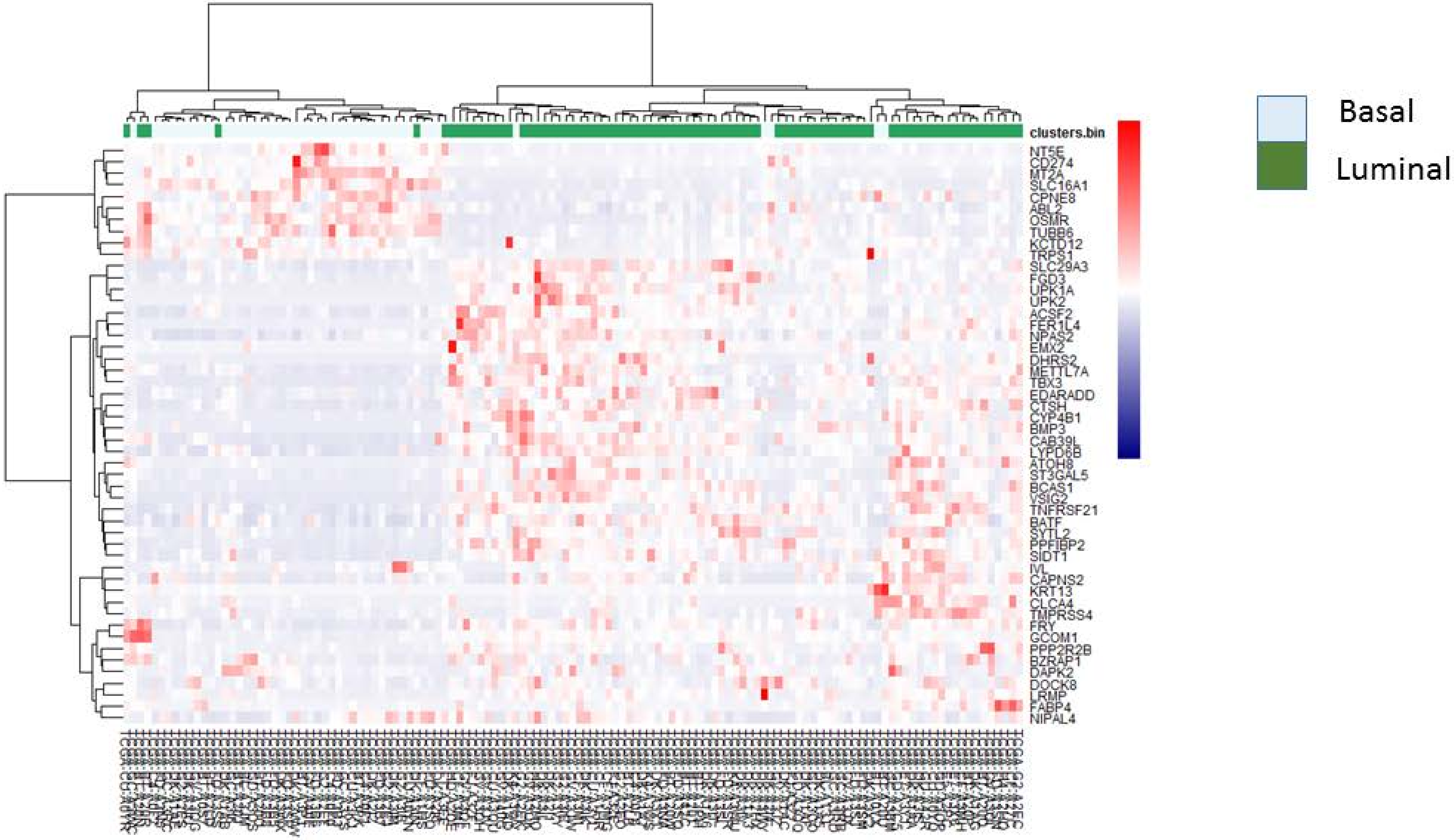
clusters.bin
NT5E
CD274
MT2A
SLC16A1
CPNE8
ABL2
OSMR
TUBB6
KCTD12
TRPS1
SLC29A3
FGD3
UPK1A
UPK2
ACSF2
FER1L4
NPAS2
EMX2
DHRS2
METTL7A
TBX3
EDARADD
CTSH
CYP4B1
BMP3
CAB39L
LYPD6B
ATOH8
ST3GAL5
BCAS1
VSIG2
TNFRSF21
BATF
SYTL2
PPFIBP2
SIDT1
IVL
CAPNS2
KRT13
CLCA4
TMPRSS4
FRY
GCOM1
PPP2R2B
BZRAP1
DAPK2
DOCK8
LRMP
FABP4
NIPAL4
Basal
Luminal

Figure S2

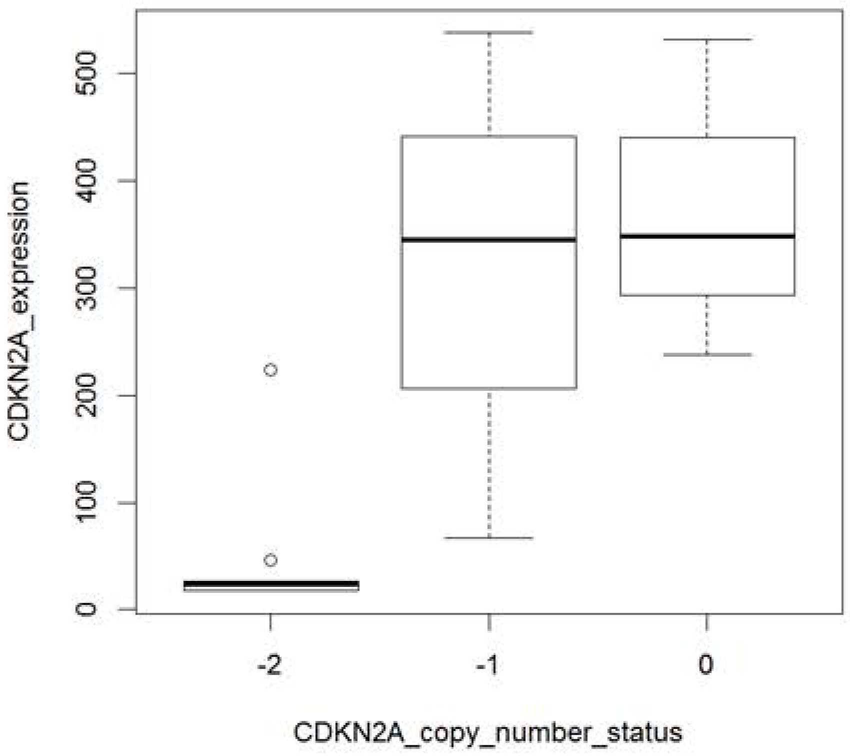
CDKN2A_expression
0
100
200
300
400
500
-2
-1
0
CDKN2A_copy_number_status

Figure S3

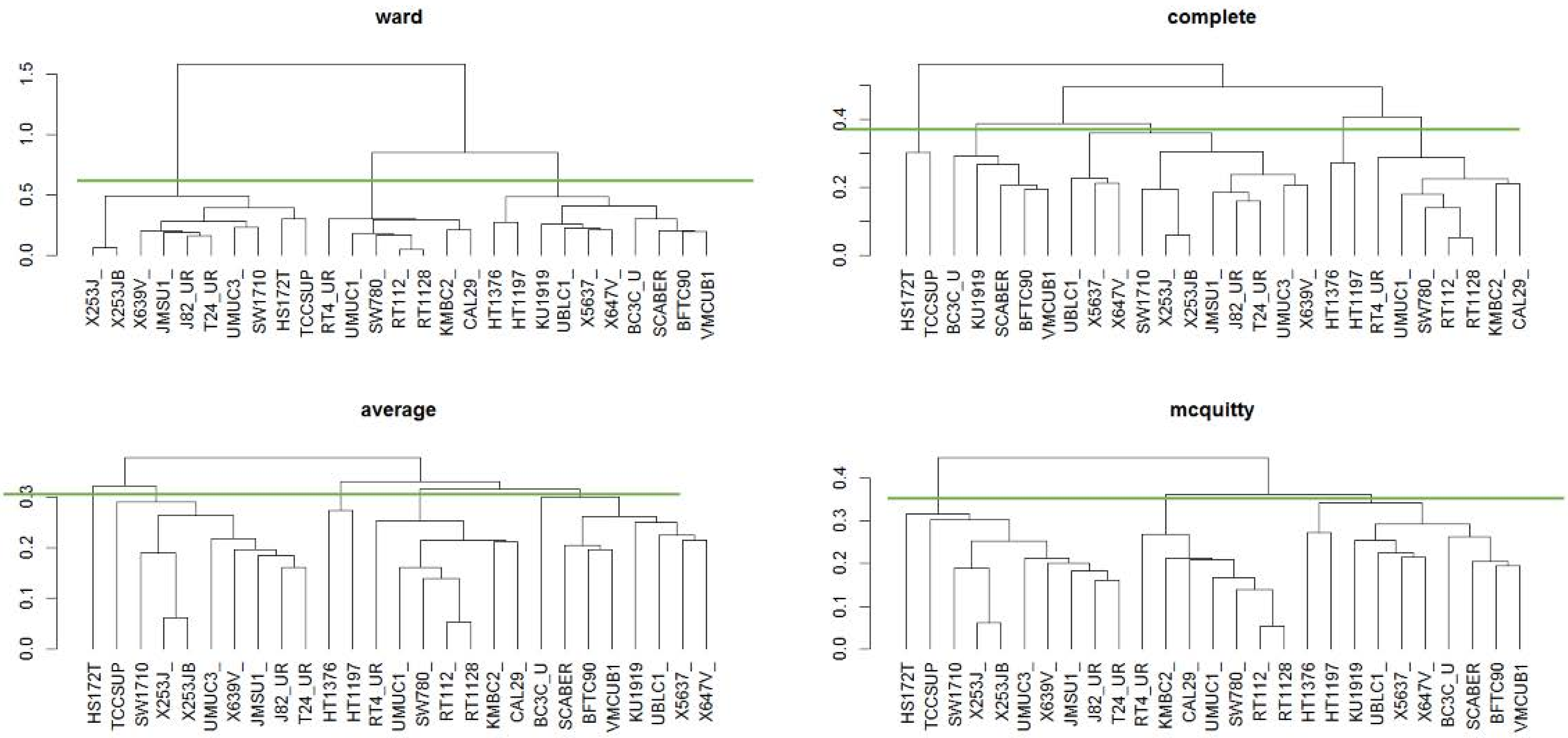
ward
0.0
0.5
1.0
1.5
X253J_
X253JB
X639V_
JMSU1_
J82_UR
T24_UR
UMUC3_
SW1710
HS172T
TCCSUP
RT4_UR
UMUC1_
SW780_
RT112_
RT1128
KMBC2_
CAL29_
HT1376
HT1197
KU1919
UBLC1_
X5637_
X647V_
BC3C_U
SCABER
BFTC90
VMCUB1
complete
0.0
0.2
0.4
HS172T
TCCSUP
BC3C_U
KU1919
SCABER
BFTC90
VMCUB1
UBLC1_
X5637_
X647V_
SW1710
X253J_
X253JB
JMSU1_
J82_UR
T24_UR
UMUC3_
X639V_
HT1376
HT1197
RT4_UR
UMUC1_
SW780_
RT112_
RT1128
KMBC2_
CAL29_
average
0.0
0.1
0.2
0.3
HS172T
TCCSUP
SW1710
X253J_
X253JB
UMUC3_
X639V_
JMSU1_
J82_UR
T24_UR
HT1376
HT1197
RT4_UR
UMUC1_
SW780_
RT112_
RT1128
KMBC2_
CAL29_
BC3C_U
SCABER
BFTC90
VMCUB1
KU1919
UBLC1_
X5637_
X647V_
mcquitty
0.0
0.1
0.2
0.3
0.4
HS172T
TCCSUP
SW1710
X253J_
X253JB
UMUC3_
X639V_
JMSU1_
J82_UR
T24_UR
RT4_UR
KMBC2_
CAL29_
UMUC1_
SW780_
RT112_
RT1128
HT1376
HT1197
KU1919
UBLC1_
X5637_
X647V_
BC3C_U
SCABER
BFTC90
VMCUB1

Figure S4

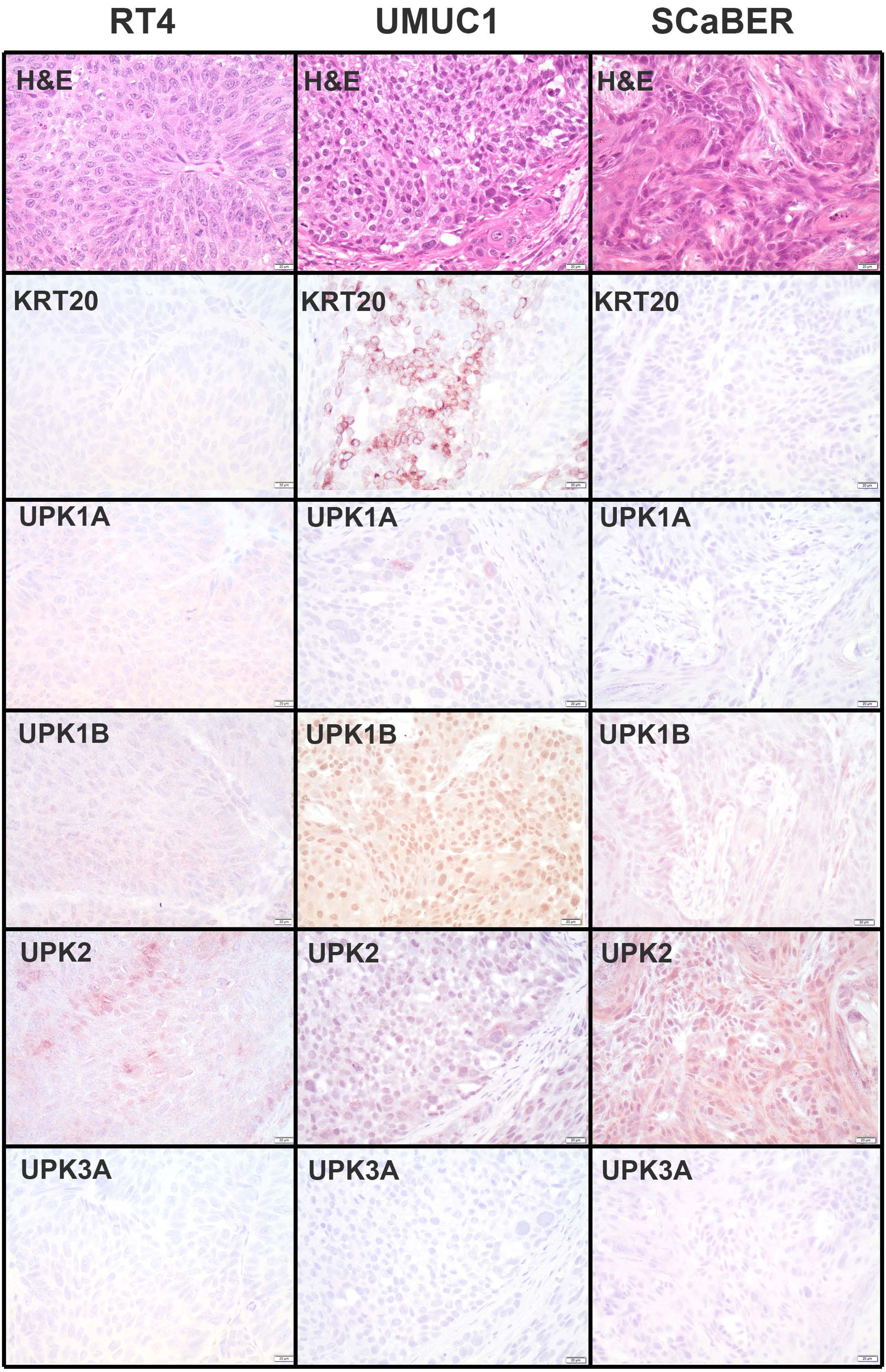
RT4
UMUC1
SCaBER
H&E
H&E
H&E
KRT20
KRT20
KRT20
UPK1A
UPK1A
UPK1A
UPK1B
UPK1B
UPK1B
UPK2
UPK2
UPK2
UPK3A
UPK3A
UPK3A

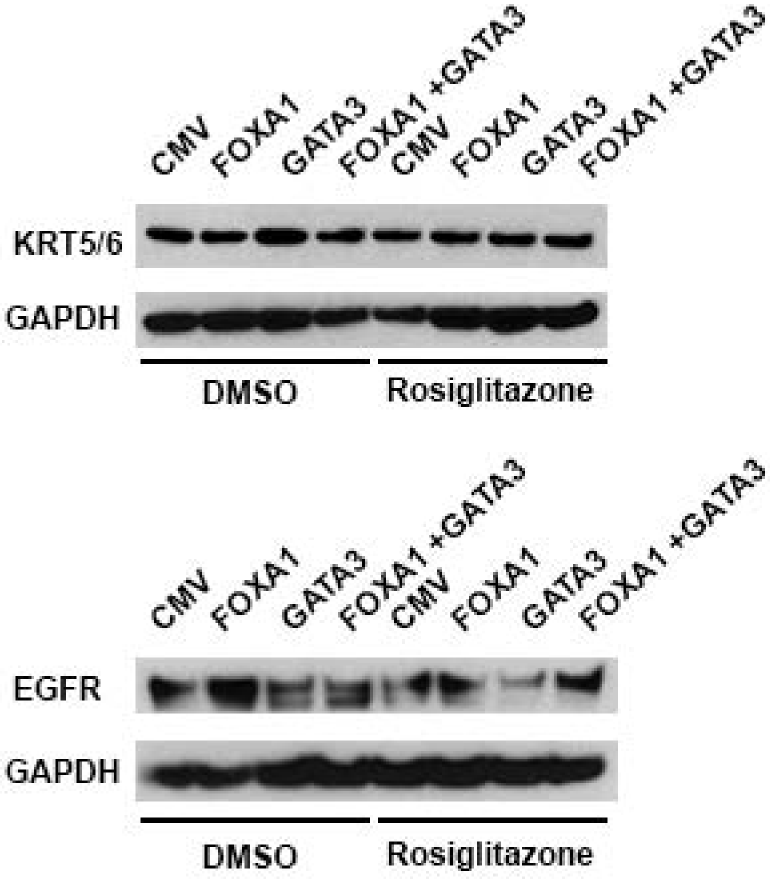
CMV
FOXA1
GATA3
FOXA1 +GATA3
CMV
FOXA1
GATA3
FOXA1 +GATA3
KRT5/6
GAPDH
DMSO
Rosiglitazone
CMV
FOXA1
GATA3
FOXA1 +GATA3
CMV
FOXA1
GATA3
FOXA1 +GATA3
EGFR
GAPDH
DMSO
Rosiglitazone

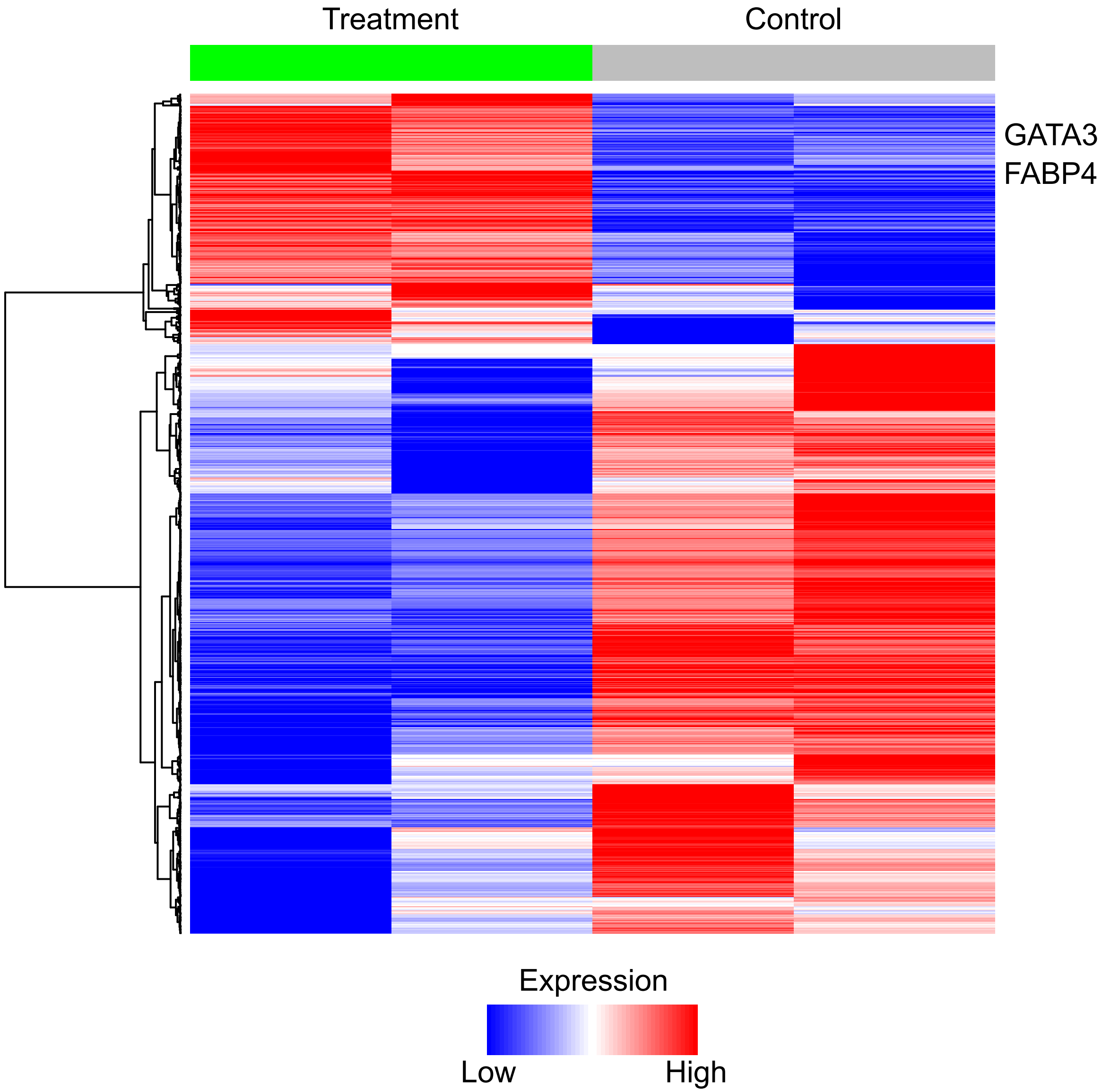
Treatment
Control
GATA3
FABP4
Expression
Low
High

Figure S7

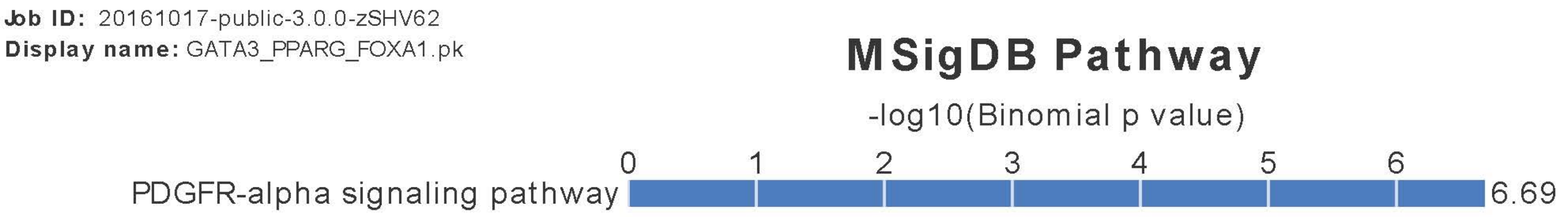

Job ID: 20161017-public-3.0.0-zSHV62
Display name: GATA3_PPARG_FOXA1.pk
MSigDB Pathway
-log10(Binomial p value)
0
1
2
3
4
5
6
PDGFR-alpha signaling pathway
6.69

Figure S8

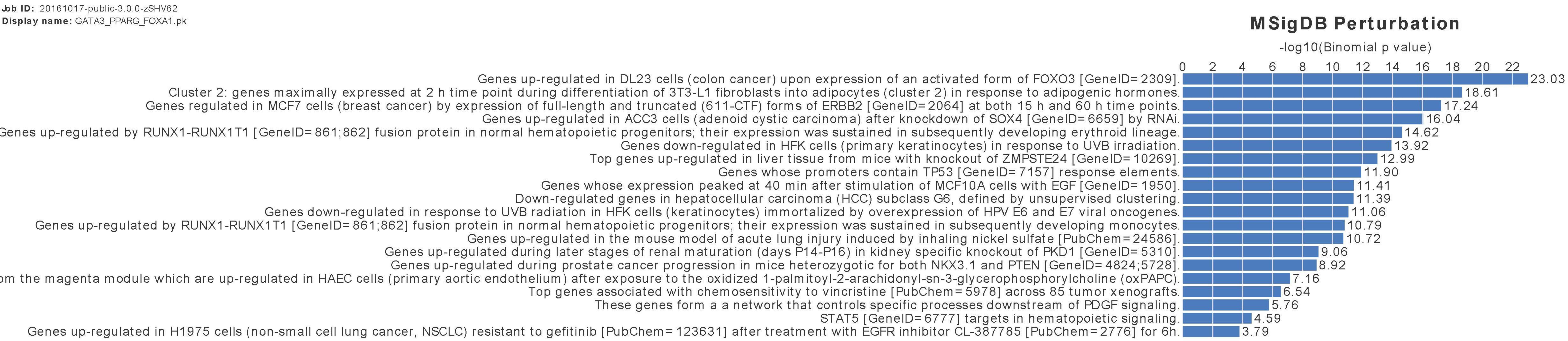
Job ID: 20161017-public-3.0.0-zSHV62
Display name: GATA3_PPARG_FOXA1.pk
MSigDB Perturbation
-log10(Binomial p value)
0 2 4 6 8 10 12 14 16 18 20 22
Genes up-regulated in DL23 cells (colon cancer) upon expression of an activated form of FOXO3 [GeneID=2309]. 23.03
Cluster 2: genes maximally expressed at 2 h time point during differentiation of 3T3-L1 fibroblasts into adipocytes (cluster 2) in response to adipogenic hormones. 18.61
Genes regulated in MCF7 cells (breast cancer) by expression of full-length and truncated (611-CTF) forms of ERBB2 [GeneID=2064] at both 15 h and 60 h time points. 17.24
Genes up-regulated in ACC3 cells (adenoid cystic carcinoma) after knockdown of SOX4 [GeneID=6659] by RNAi. 16.04
Genes up-regulated by RUNX1-RUNX1T1 [GeneID=861;862] fusion protein in normal hematopoietic progenitors; their expression was sustained in subsequently developing erythroid lineage. 14.62
Genes down-regulated in HFK cells (primary keratinocytes) in response to UVB irradiation. 13.92
Top genes up-regulated in liver tissue from mice with knockout of ZMPSTE24 [GeneID=10269]. 12.99
Genes whose promoters contain TP53 [GeneID=7157] response elements. 11.90
Genes whose expression peaked at 40 min after stimulation of MCF10A cells with EGF [GeneID=1950]. 11.41
Down-regulated genes in hepatocellular carcinoma (HCC) subclass G6, defined by unsupervised clustering. 11.39
Genes down-regulated in response to UVB radiation in HFK cells (keratinocytes) immortalized by overexpression of HPV E6 and E7 viral oncogenes. 11.06
Genes up-regulated by RUNX1-RUNX1T1 [GeneID=861;862] fusion protein in normal hematopoietic progenitors; their expression was sustained in subsequently developing monocytes. 10.79
Genes up-regulated in the mouse model of acute lung injury induced by inhaling nickel sulfate [PubChem=24586]. 10.72
Genes up-regulated during later stages of renal maturation (days P14-P16) in kidney specific knockout of PKD1 [GeneID=5310]. 9.06
Genes up-regulated during prostate cancer progression in mice heterozygotic for both NKX3.1 and PTEN [GeneID=4824;5728]. 8.92
om the magenta module which are up-regulated in HAEC cells (primary aortic endothelium) after exposure to the oxidized 1-palmitoyl-2-arachidonyl-sn-3-glycerophosphorylcholine (oxPAPC). 7.16
Top genes associated with chemosensitivity to vincristine [PubChem=5978] across 85 tumor xenografts. 6.54
These genes form a a network that controls specific processes downstream of PDGF signaling. 5.76
STAT5 [GeneID=6777] targets in hematopoietic signaling. 4.59
Genes up-regulated in H1975 cells (non-small cell lung cancer, NSCLC) resistant to gefitinib [PubChem=123631] after treatment with EGFR inhibitor CL-387785 [PubChem=2776] for 6h. 3.79

Figure S9

Job ID: 20161017-public-3.0.0-zSHV62
Display Name: GATA3_PPARG_FOXA1.pk

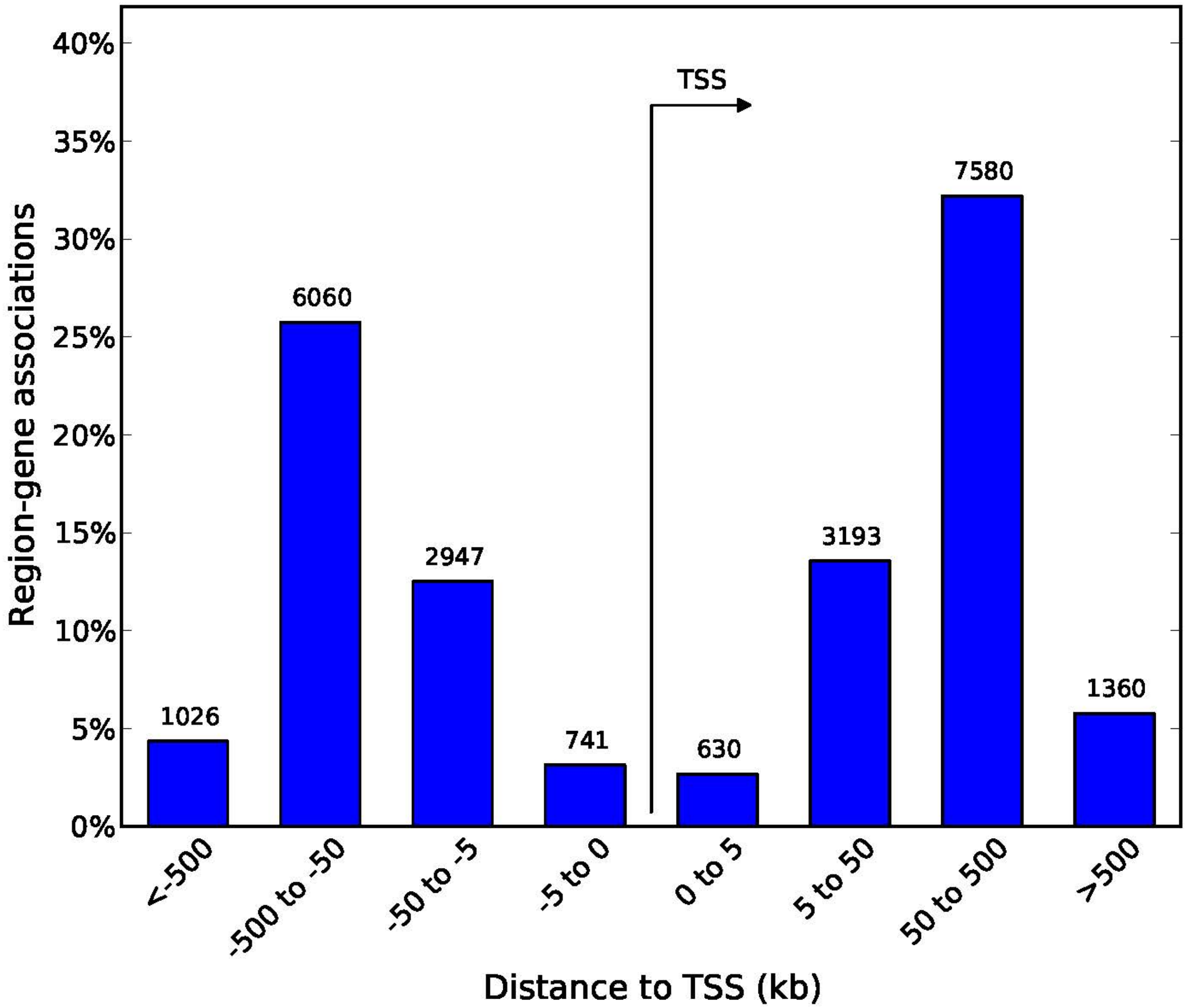

Supplement: Supplemental Methods and Data [file srep38531-s1.pdf]
